# Altered capsaicin levels in domesticated chili pepper varieties affect the interaction between a generalist herbivore and its ectoparasitoid

**DOI:** 10.1007/s10340-021-01399-8

**Published:** 2021-06-25

**Authors:** Yosra Chabaane, Carla Marques Arce, Gaëtan Glauser, Betty Benrey

**Affiliations:** 1 Laboratory of Evolutionary Entomology, Institute of Biology, University of Neuchâtel, Rue Emile-Argand 11, 2000 Neuchâtel, Switzerland; 2 Fundamental and Applied Research in Chemical Ecology, Institute of Biology, University of Neuchâtel, Rue Emile-Argand 11, 2000 Neuchâtel, Switzerland; 3 Neuchâtel Platform of Analytical Chemistry, Institute of Chemistry, University of Neuchâtel, Rue Emile-Argand 11, 2000 Neuchâtel, Switzerland

**Keywords:** Chili pepper, Domestication, Capsaicinoids, Plant-mediated, Sequestration, Tritrophic interactions

## Abstract

Plant domestication has commonly reduced levels of secondary metabolites known to confer resistance against insects. Chili pepper is a special case because the fruits of different varieties have been selected for lower and higher levels of capsaicin, the main compound associated with defence. This may have important consequences for insect herbivores and their natural enemies. Despite the widespread consumption of chili peppers worldwide, the effects of capsaicin on insects are poorly understood. Here, we investigated the effect of capsaicin on a generalist herbivore, *Spodoptera latifascia* (Lepidoptera: Noctuidae) and its ectoparasitoid, *Euplectrus platyhypenae* (Hymenoptera: Eulophidae). Using chili varieties with three pungency levels: non-pungent (Padron), mild (Cayenne) and highly pungent (Habanero), as well as artificial diets spiked with three different levels of synthetic capsaicin, we determined whether higher capsaicin levels negatively affect the performance of these insects. Overall, capsaicin had a negative effect on both herbivore and parasitoid performance*,* particularly at high concentrations. Caterpillars reared on highly pungent fruits and high-capsaicin diet had longer development time, reduced pupation success, lower adult emergence, but also lower parasitism rates than caterpillars reared on mild or non-capsaicin treatments. In addition, we found that the caterpillars were capable of sequestering capsaicinoids in their haemolymph when fed on the high pungent variety with consequences for parasitoids’ performance and oviposition decisions. These results increase our understanding of the role of capsaicin as a chemical defence against insects and its potential implications for pest management.

**Supplementary Information:**

The online version contains supplementary material available at 10.1007/s10340-021-01399-8.

## Key message

- Domestication of chili peppers has altered the content of capsaicin in fruits, which is responsible for the chili’s pungency.
- Capsaicin has been associated with defense against insects, but the evidence is limited.
- We tested the effects of capsaicin on a generalist herbivore and its parasitoid.
- Capsaicin had negative effects on herbivore performance, cascading up to the parasitoid.
- The results support the role of capsaicin as a chemical defense against insects, with possible implications for pest management.

## Introduction

Plant domestication has resulted in a suite of morphological, nutritional and chemical traits that distinguish crops from their wild counterparts (Gepts 2004; Smartt and Simmonds 1995). One of the main changes in crops is a reduction in secondary metabolites (Meyer et al. 2012). The reason for this change is to render plants more suitable for human consumption (Ladizinsky 2012). In this context, chili pepper (*Capsicum* spp., family Solanaceae) offers a unique model to examine the relationship between domestication and altered levels of chemical defences.

The genus *Capsicum* is resolved as a monophyletic group with five domesticated taxa and around 20–30 wild species (Carrizo García et al. 2016). In contrast to most crop plants for which domestication has resulted in a decrease of secondary metabolites, chili peppers have been selected for both increased and decreased levels of their main secondary metabolites so-called capsaicinoids (Aza-González et al. 2011; Kim 2014) as compared to the wild ancestor, Chiltepin (González-Zamora et al. 2015; Tewksbury et al. 2008). These secondary compounds are biosynthesized and accumulated in the placenta tissue and responsible for the pungency or spiciness in chili fruits (Pickersgill 2016). The varietal selection was performed along a gradient from low to high pungency (from 0 to 1,500,000 Scoville units) (Scoville 1912). The main capsaicinoids are capsaicin and dihydrocapsaicin which represent 90% of the whole capsaicinoids in fruits (Govindarajan and Salzer 1985). Capsaicinoids are synthetized via two different pathways, the phenylpropanoid and the branched-chain fatty acid pathways (Aza-González et al. 2011), and two genes were identified to be responsible for chili pepper pungency, Pun1 and pAMT located, respectively, on chromosome 2 and chromosome 3 (Lang et al. 2006; Stewart Jr et al. 2005). The unfunctional alleles of these genes cause the loss of pungency found in sweet pepper varieties (Tsurumaki and Sasanuma 2019).

Capsaicinoids are known to have deterrent and medicinal properties for mammals. For example, chili has been used as a crop guarding system in different African and Asian countries to reduce human–elephant conflicts (Chang'a et al. 2016; Hedges and Gunaryadi 2010). Interestingly, birds are not sensitive to capsaicin (Mason and Maruniak 1983; Szolcsányi et al. 1986). A comparison between chicken capsaicin vanilloid receptors (cTRPV19) and its rat counterparts (rTRPV1) showed a high structural divergence (only 68% amino acid identity) (Jordt and Julius 2002). The difference between both vanilloid receptors might be the result of selective pressures that facilitate the differentiation of the ecological niche of each species. Indeed, it has been suggested that birds evolved as vectors for fruit dispersion whereas mammals were repelled to avoid the destruction of seeds (Tewksbury and Nabhan 2001; Tewksbury et al. 2008). Chili has been also known for its medicinal uses long before the Spanish colonization of the Americas and since the Mayan civilization (Cichewicz and Thorpe 1996; Pickersgill 2016; Witting et al. 2000).

The effect of capsaicin on pathogenic bacteria and fungi has also been widely studied. Capsaicin inhibits and retards the growth of several human (e.g. *Helicobacter pylori, Escherichia coli, Streptococcus pyogenes*), soil (e.g. *Bacillus subtilis* and *Pseudomonas solanacearum)* and plant pathogenic (e.g. *Xanthomonas campestris*, *Pseudomonas syringae*) bacteria (Argaez et al. 2009; Jones et al. 1997; Marini et al. 2015; Molina-Torres 1999). For example, non-pungent wild chili fruits were twice more infested by *Fusarium spp.* as compared to wild pungent fruits, suggesting that capsaicinoids protect fruits from pathogenic fungi (Haak et al. 2012; Tewksbury et al. 2008). Likely, the reason why hot chilies have been used for food preservation in many regions, long before the use of refrigerators (Omolo 2014).

For insects, there is a common assumption that capsaicin is toxic. Several studies have shown that capsaicin deters oviposition (Cowles et al. 1989), slows down larval development (Ahn et al. 2011a; Weissenberg et al. 1986) and inhibits feeding (Hori et al. 2011). Moreover, synthetic capsaicin has even been used as a pesticide against some insect pests (wilsonKoleva-Gudeva et al. 2013; Wilson 1996). Although the above studies demonstrate the negative effects of capsaicin on insect herbivores, they were all conducted using either artificial diet in which pure capsaicin or dried chili powder was added. As yet, only a handful study have examined the effect of capsaicin on insect herbivores using fresh fruits (Tęgowska et al. 2005).

Plant secondary metabolites can also affect the natural enemies of herbivores in different ways (Chen et al. 2015; Turlings and Benrey 1998). For example, parasitoid wasps can benefit from the volatiles emitted from herbivore-damaged plants and use them as cues to find their host (Turlings et al. 1990; Vet and Dicke 1992). In addition, secondary metabolites can slow down herbivore development and thus increase their time of exposure to natural enemies (Benrey and Denno 1997; Price et al. 1980). Alternatively, some herbivores can sequester and store plant toxins in their bodies making them unpalatable or toxic for their natural enemies (Opitz and Müller 2009; Rowell-Rahier and Pasteels 1992). For example, El‐Heneidy et al. (1988) found that the survival of an Ichneumonid parasitoid (*Hyposoter annulipes*) (Hymenoptera: Ichneumonidae) was reduced when its larval host, the fall armyworm (*Spodoptera frugiperda*) (Lepidoptera: Noctuidae), was fed on artificial diet mixed with nicotine. In another study, it was shown that by sequestering alkaloids from its host plant, larvae of the sawfly (*Rhadinoceraea nodicornis*) (Hymenoptera, Tenthredinidae) were protected against generalist predators (Schaffner et al. 1994). To date, however, the effects of capsaicin on the natural enemies of herbivores are not known. Knowing whether capsaicin has a negative effect on insect herbivores and these effects cascade up to their natural enemies is a valuable information for pest management practices of chili pepper.

The aim of this study was to examine how altered capsaicin levels in chili peppers as a result of varietal selection affect the tritrophic interaction with a generalist herbivore *Spodoptera latifascia* (Lepidoptera: Noctuidae) and one of its larval ectoparasitoid, *Euplectrus platyhypenae* (Hymenoptera: Eulophidae). We used a combination of chemical analyses and behavioural assays to address the following questions: (1) is capsaicin toxic for these insects? and (2) what are the direct and indirect (via the host caterpillar) effects of capsaicin on herbivore and parasitoid performance? To answer these, first we determined capsaicin levels in fruits of three varieties selected for different pungency levels. Secondly, we reared herbivores on fruits of these varieties, as well as parasitoids on caterpillars fed with these fruits and determined their performance, and finally, we reared parasitized and non-parasitized caterpillars on artificial diet with different levels of synthetic capsaicin.

Our results provide insight into how varietal selection of this important crop has influenced its interactions with herbivores and their parasitoids. Furthermore, to our knowledge, this is the first time that the effects of capsaicin on the third trophic level are examined.

## Material and methods

### Chili fruits

Chili fruits were purchased from a local market in Neuchâtel, Switzerland. We selected three varieties based on their known pungency level: non-pungent variety Padron (*C. annuum*), mild variety Cayenne (*C. annuum*) and highly pungent variety, Habanero (*C. chinense*). These varieties are originally from Latin America (Muñoz-Ramírez et al. 2018), except for Padron, which was selected in Galicia, Spain (Katz 2009). These varieties were used in all the experiments with fruits.

### Insects

*Spodoptera latifascia*, commonly known as the Velvet armyworm, occurs naturally throughout Mexico and Central America (Saunders et al. 1988; Zagatti et al. 1995). It is a polyphagous insect whose host range includes several crops such as potato, cotton, soybeans, maize, and beans (Cuny et al. 2018; Habib et al. 1982). Larvae have been frequently found feeding on leaves of chili plants in Mexico (Traine et al. 2020).

*Euplectrus platyhypenae* is a gregarious koinobiont ectoparasitoid, originated from Mexico, and parasitizes third and fourth instars of Noctuid and Geometrid caterpillars (Muniappan et al. 2004; Murúa and Virla 2004; Swezey 1924). Prior to oviposition, female wasps inject a venom on the dorsum of the caterpillar to inhibit molting without killing their host. One female lays up to 20 eggs that develop on the dorsal segments of the host’s body feeding on its haemolymph (Coudron et al. 1990; Nakamatsu and Tanaka 2003). Before pupation, the parasitoid larvae use their saliva to kill the host and move underneath the cadaver to pupate (Nakamatsu and Tanaka 2004). Approximately one-week later adults emerge (Nakamatsu and Tanaka 2003).

Colonies of *S. latifascia* and *E. platyhypenae* were established with insects originally collected from beans, squash, and chili pepper in the experimental campus of the Universidad del Mar, in Puerto Escondido, (Oaxaca, Mexico; 15°55′33.3″N, 97°09′03.0″W). Then, they were reared at the University of Neuchâtel under quarantine conditions, level 3 (26 °C, 60% relative humidity and L12:D12). Caterpillars were fed on artificial diet (soy-wheat germ diet, Frontier scientific services, USA) in plastic boxes (13 × 15 × 5 cm) with fabric mesh for aeration. Parasitoids were reared on two different species, *S. latifascia* and *S. frugiperda* third instar caterpillars, which were fed with artificial diet and maize leaves were offered to maximize caterpillar survival until parasitoid pupation. Parasitoid adults were kept in 30 × 30 × 30 cm mesh cages (Bioquip Products) with water and honey as food source.

### Quantification of capsaicinoids in chili fruits and herbivore haemolymph

To verify the pungency level of the fruits of the selected varieties, we quantified the capsaicinoids content in chili fruits. Whole fruits were oven dried for 48 h at 60 °C following the method developed by Collins et al. (1995). Once dried, each fruit was ground separately with a mortar to obtain a fine powder and 10 mg was extracted with 1 ml of methanol. The mixture was then centrifuged for 5 min at 14 000 rpm, 700 ul of supernatant was collected and further diluted 100′000-fold prior to HPLC analysis. For each variety, we had 10 replicates (1 replicate = 1 fruit).

To investigate whether capsaicinoids can be sequestered to other tissues apart from the gut, we measured the capsaicin content in the haemolymph of *S. latifascia* caterpillars fed for 7 days on the three chili fruit varieties (non-pungent variety Padron, mild variety Cayenne and highly pungent variety Habanero). The haemolymph was collected by puncturing the cuticle at the dorsal part of the thorax. From each larva, we collected 2 µl of haemolymph exuded immediately after the incision in an Eppendorf tube containing 10 µl of an anticoagulant solution composed of 98 mM NaOH, 186 mM NaCl, 17 mM Na2EDTA and 41 mM citric acid (pH 4.5) (Haine et al. 2007). We had five replicates per treatment and each replicate was a pool of two caterpillars. Then, 400 µl of methanol (100%) was added to each sample that were centrifuged for 5 min at 14 000 rpm and the supernatants were filtered using a hydrophilic PTFE filter (size = 13 mm; Thermo Fisher Scientific) and a single-use-syringe (1 ml, Soft Ject). The samples were diluted 1000-fold with methanol (100%) prior to analysis and they were kept at − 80 °C until further analysis.

The capsaicinoid content was analysed using an Acquity ultra-high-pressure liquid chromatography (UHPLC) system coupled to a Synapt G2 QTOF mass spectrometer (Waters, Milford, MA, USA) controlled by Masslynx 4.1. The separation was performed on a Waters Acquity BEH C18 column (50 × 2.1 mm i.d., 1.7 μm particle size) thermostated at 25 °C. Mobile phases consisted of water containing 0.05% formic acid (solvent A) and acetonitrile containing 0.05% formic acid (solvent B). Standards of capsaicin (> 95% from *Capsicum* spp,) and dihydrocapsaicin (> 85% from *capsicum* spp.) from Sigma-Aldrich (St. Louis, Missouri) were used to identify and quantify capsaicinoids in fruits and in the haemolymph of *S. latifascia* caterpillars. Standard curves were prepared using concentrations of 0.04, 0.2, 1 and 5 ug/ml.

### Bioassays

To investigate the impact of capsaicin on the performance of the herbivore *S. latifascia* and the parasitoid *E. platyhypenae,* we fed caterpillars separately on chili fruits and capsaicin-spiked artificial diet.

### Effect of pungency level on the performance of the herbivore *Spodoptera latifascia*

Caterpillars were fed on mature chili fruits from three different varieties: non-pungent variety Padron, mild variety Cayenne and highly pungent variety Habanero. Three-day-old larvae were individually placed, with a piece of fruit containing the placenta part, in a small cylindrical plastic container (0.23 L) covered with mesh for aeration. New fresh fruits were provided for larvae every other day. The number of replicates (plastic containers) used for each variety is as follows: non-pungent = 20, mild = 18, and highly pungent = 17. The parameters recorded were: caterpillars weight until they pupate, pupation (number of pupae/number of remaining larvae) *100), pupal weight measured 24 h after pupation, and adult emergence (number of adults/number of remaining larvae)*100). The measurements were taken every three days for caterpillar weight until pupation and then every day until adult emergence. Larval and pupal weights were measured using an electronic balance (BP 161P, Sartorius, Goettingen, Germany).

To determine the effect of capsaicin alone, without other possible effects of the chili fruits, we conducted a parallel experiment with artificial diet (Soy-wheat germ diet, Frontier scientific services) spiked with capsaicin. Larvae were reared on diet with three different levels of synthetic capsaicin to mimic the gradient of pungency used for the chili fruit experiment: control (without capsaicin), low-capsaicin (20 ppm = 0,02 mg g^−1^) and high-capsaicin diet (200 ppm = 0,2 mg g^−1^). The number of replicates used for each capsaicin treatment is as follows: no-capsaicin = 17, 20 ppm = 19, and 200 ppm = 18. The capsaicin-spiked diet for the feeding experiment was prepared by adding two different concentrations of capsaicin (≥ 95% from *Capsicum* spp. from Sigma-Aldrich, Switzerland) dissolved in ethanol and mixed at 20 ppm and 200 ppm with the diet before solidification. For the control treatment, only ethanol was added. Due to the very irritating nature of pure capsaicin, we could not mimic the exact levels as found in Habanero fruits. However, we used concentrations of capsaicin that have been proven effective in other studies with Noctuidae species (Ahn et al. 2011a). We carried out this experiment using the same protocol as for the experiment with fruits (Sect. 1a).

### Effect of pungency level on the performance of the parasitoid *Euplectrus platyhypenae*

To investigate whether the pungency level in chili fruit affects the third trophic level, the performance of *E. platyhypenae* was assessed when reared on *S. latifascia* larvae fed on the three chili varieties (Padron = non-pungent, Cayenne = mild and Habanero = highly pungent). For the control treatment (*N* = 10), larvae were fed on a maize leaf with a piece of artificial diet to assure optimal oviposition by the wasp *E. platyhypenae* (Traine et al. 2020).

Caterpillars were reared individually for 6 days on the three varieties. On day seven, one fourth-instar caterpillar was placed on a piece of fruit from its rearing variety and placed in a 9 × 2 cm Petri dish. Subsequently, one couple of *E. platyhypenae* were introduced in the Petri dish. The food source for the caterpillars (host) was present during the whole period of exposure to the parasitoids. Thirteen replicates (Petri dishes) were used for each variety. The average time for parasitism was between three and four days. As soon as the first clutch of eggs was observed on the larvae, the adults of *E. platyhypenae* were removed. Afterward, we recorded parasitism (number of parasitized larvae/number of remaining larvae)*100) and the clutch size defined as the number of eggs laid in a single reproductive bout (Godfray 1994). For each parasitized larva, the number of eggs laid by the wasp was counted using a hand lens (Triplet, 30X-21 mm).

In a parallel experiment, we followed the same procedure but removed the fruits and replaced them with no-capsaicin artificial diet when the caterpillars (host) were exposed to the parasitoids. This allowed us to test whether female wasps were capable of perceiving the capsaicin present in the host haemolymph. We recorded the same parameters as in the previous experiment. Five replicates (Petri dishes) were used for each variety.

The goal of this experiment was to determine the effect of capsaicin alone on the parasitoid response independent of other potential fruit factors. We used artificial diet spiked with capsaicin at three different concentrations 0, 20 and 200 ppm. The same protocol as for the experiment with fruits was used. In a first experiment, larvae were fed on their original capsaicin-spiked diet (capsaicin diet) before and after the exposure to the wasps. In a second experiment, the capsaicin-spiked diet was replaced by a common diet without capsaicin (no-capsaicin diet) only when caterpillars were exposed to the wasp. The purpose of these two different designs was to examine whether female wasps can perceive the capsaicin present in the host haemolymph independently when fruits were not present. In both experiments, five replicates (Petri dishes) were used for each capsaicin treatment.

### Statistical analysis

All statistical analyses were performed in R statistical software (version 3.5.3; R Development Core Team 2020) by using ANOVA, followed by residual analysis to verify suitability of distributions of the tested models. To test the effect of capsaicin on caterpillars’ weight, when feeding on either chili fruits or on artificial diet spiked with synthetic capsaicin, generalized linear mixed models (GLMMs) with a Gaussian distribution were used. GLMMs included ‘treatment’, ‘time’ and the interactions between ‘treatment’ and ‘time’, replicate and time as random factors. Least squares means (*LSMeans)* were used to compare significantly differences among treatments. Generalized linear models (GLMs) with a Gaussian distribution were used to verify the pupal weight, parasitoid clutch size, capsaicinoid contents in fruits and haemolymph. Least squares means (*LSMeans)* were used to compare significantly differences among treatments. Parasitoid emergence, herbivore pupation rate and adult emergence were analysed using generalized linear models (GLM) under binomial distribution. The effects of treatments on caterpillar’s pupation time were analysed using the package “Survival” from R under Weibull distribution. The overdispersion of the data was verified and if necessary, the correction by using quasibinomial was applied. The sample size and number of replicates for all experiments are indicated directly in figure captions.

## Results

### Quantification of capsaicinoid in fruits

**Fig. 1:**
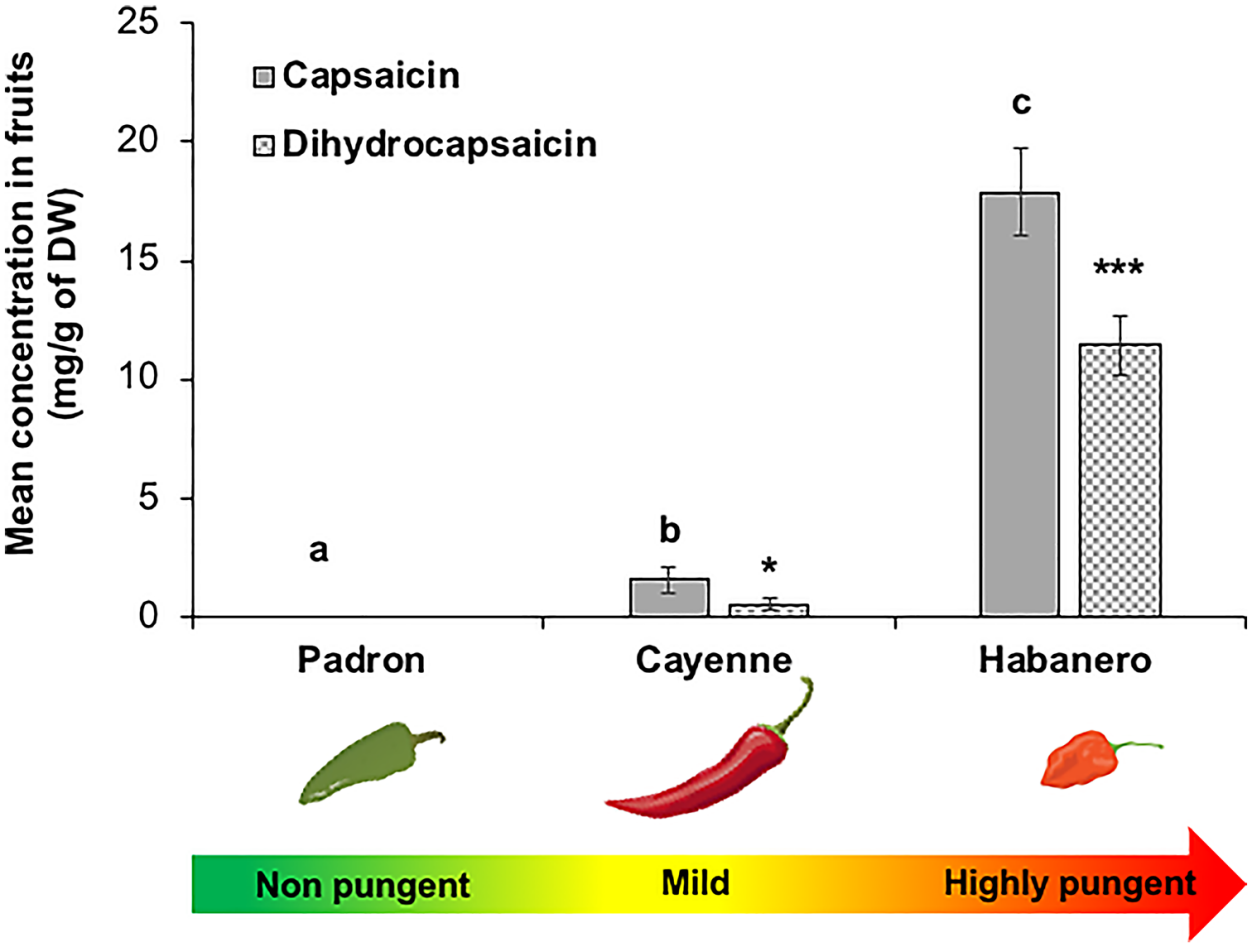
Mean (± SEM) capsaicinoids content (mg/g of DW) in the three chili varieties, Padron, Cayenne and Habanero. Difference among treatments is indicated by different letters for capsaicin and stars for the dihydrocapsaicin concentrations (F-test, Tukey post hoc test with Bonferroni correction: *P* < 0.001, *N* = 10)

Capsaicinoid analysis of the chili fruits showed considerable quantitative variation among the three chili varieties both in capsaicin and dihydrocapsaicin levels (Fig. 1, capsaicin F_[2,33]_ = 79.138, d.f = 2, *P* < 0.001 and dihydrocapsaicin; F_[2,33]_ = 73.585, d.f = 2, *P* < 0.001). The Habanero variety had 11 and 22 times more capsaicin and dihydrocapsaicin (capsaicin: 17.89 mg g^−1^ of dry weight (DW); dihydrocapsaicin: 11.41 mg g^−1^ DW), respectively, than the mild Cayenne variety (capsaicin: 1.53 mg g^−1^ DW; dihydrocapsaicin: 0.51 mg g^−1^ DW), whereas the Padron variety had no capsaicinoids at all (Fig. 1). In the case of Habanero and Cayenne, capsaicin content was around 1.5 and 3 times higher than dihydrocapsaicin, respectively (Fig. 1).

### Effect of pungency level on the performance of the herbivore *Spodoptera latifascia*

**Fig. 2:**
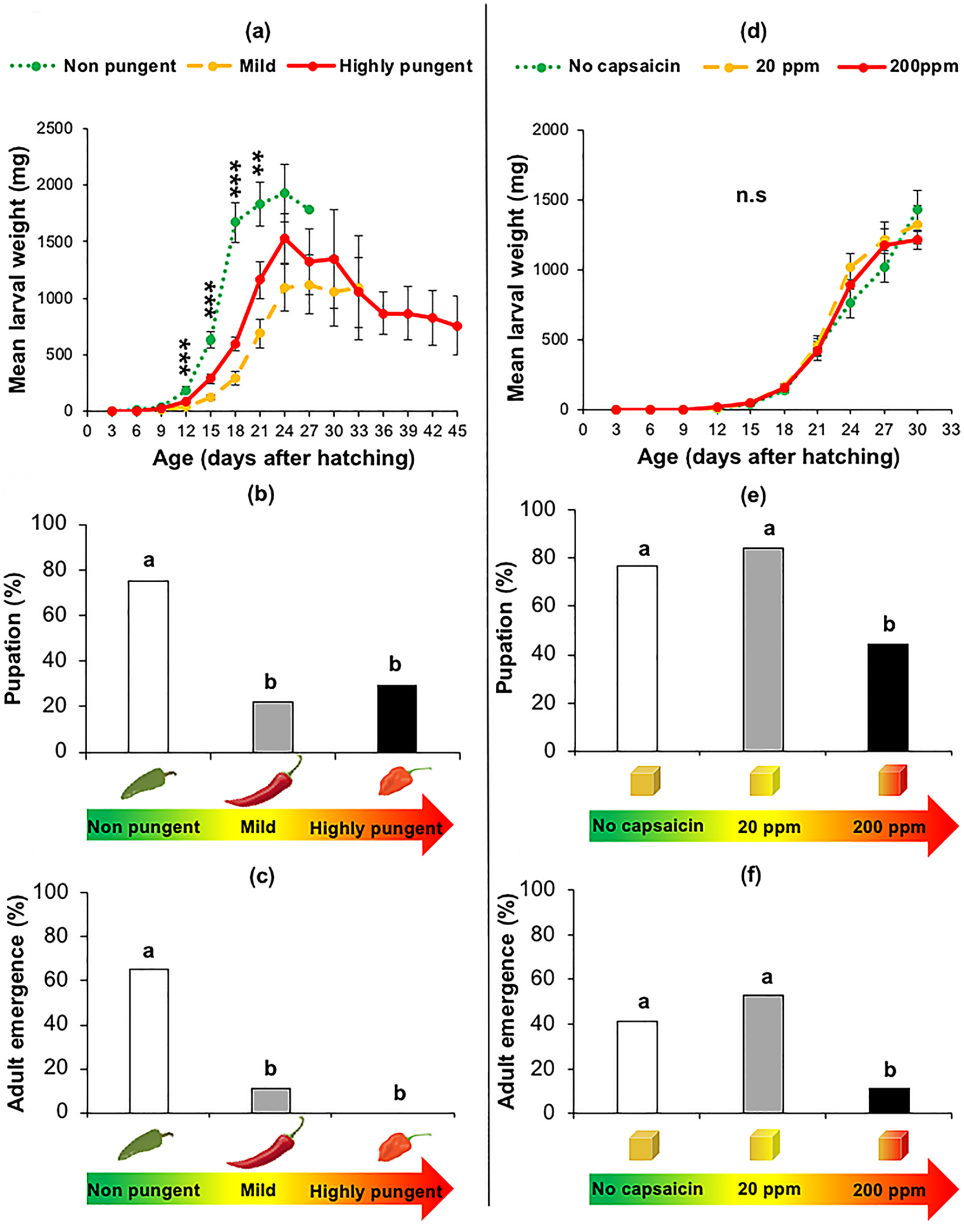
Effect of pungency level (in fruits) and capsaicin content (in diet) on the performance of *Spodoptera latifascia*: **a** mean larval weight (mg), **b** pupation (%) and **c** adult emergence (%) of *S. latifascia* feeding on chili fruits with three different pungency levels non-pungent, mild, and highly pungent, **d** mean larval weight (mg), **e** pupation (%) and **f** adult emergence (%) of caterpillars feeding on artificial diet mixed with three levels of synthetic capsaicin (no capsaicin, 20 and 200 ppm). Means in **a** for the same age capped with asterisks are significantly different (F-test, Tukey post hoc test with Bonferroni correction: ** *P* < 0.01, ***P < 0.001). Different letters indicate a significant difference between treatments for pupation and adult emergence (Chi-test, Tukey post hoc test with Bonferroni correction: *P* < 0.01). Sample sizes: Fig. 2a, b and c, non-pungent = 20, mild = 18 and highly pungent = 17; Fig. 2d, e and f, no-capsaicin = 17, 20 ppm = 19 and 200 ppm = 18

The performance of caterpillars of *S. latifascia* was negatively affected by the pungency levels in the fruits. Caterpillars grew larger (Fig. 2a, *χ*^2^ = 47.492, d.f = 2, *P* < 0.001) on the non-pungent variety than on mild and highly pungent fruits. Moreover, caterpillars took only 27 days to pupate in the non-pungent variety compared to 33 and 45 days in the mild and highly pungent varieties, respectively (Fig. 2a, *χ*^2^ = 45.95, d.f = 2, *P* < 0.001).

Feeding on mild and highly pungent varieties significantly reduced the pupation rate compared to feeding on non-pungent chili fruits (Fig. 2b, *χ*^2^ = 62.160, d.f = 2, *P* < 0.001). However, no difference among the treatments was found for the weight of pupae (Supplementary Table 1, F_[2, 21]_ = 0.1167, d.f = 2, *P* = 0.89). The percentage of adult emergence for *S. latifascia* was significantly higher on the non-pungent Padron variety than on the mild variety (Fig. 2c, *χ*^2^ = 38.213, d.f. = 2, *P* < 0.001). On the highly pungent variety, the pupae did not reach the adult stage (Fig. 2c).

Capsaicin-spiked diet did not have a significant effect on the larval weight of *S. latifascia* caterpillars (Fig. 2d, *χ*^2^ = 4.6453, d.f = 2, *P* = 0.96). However, pupation rate and adult emergence were negatively affected at the higher concentration of 200 ppm (Fig. 2e, *χ*^2^ = 60.478, d.f = 2, *P* = 0.009 and Fig. 2f, *χ*^2^ = 58.261, d.f = 1, *P* = 0.001). Caterpillars that fed on a diet containing 200 ppm of capsaicin pupated on average 35% less than caterpillars on the capsaicin-free and 20-ppm diets (Fig. 2e). Moreover, 30% and 41% fewer adults emerged from the 200-ppm diet treatment than from the no-capsaicin and 20-ppm treatments (Fig. 2f).

### Effect of pungency level on the performance of *Euplectrus platyhypenae*

**Fig. 3:**
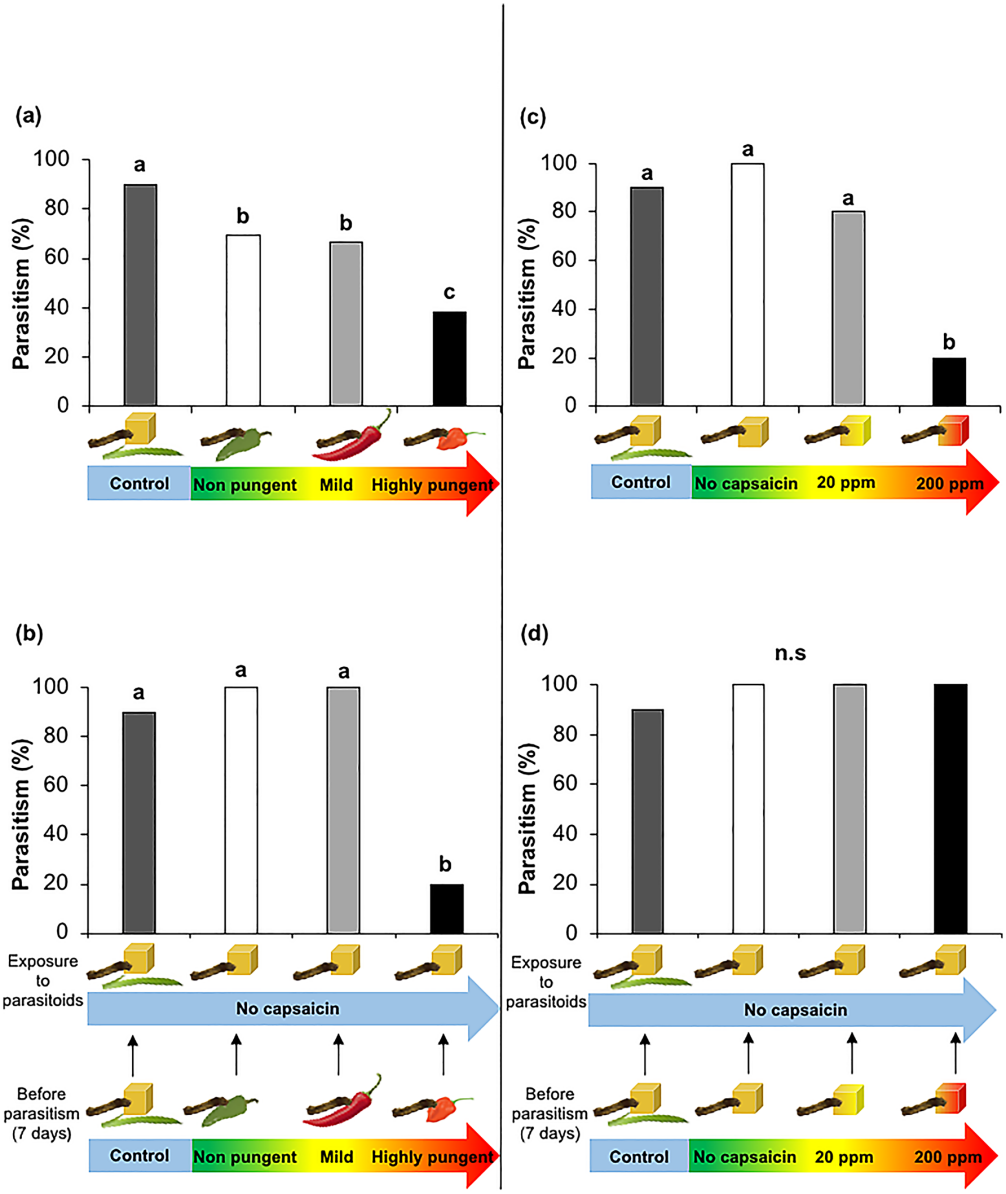
Parasitism rate (%) of *Euplectrus platyhypenae* on *Spodoptera latifascia* caterpillars feeding on **a** a control diet and on chili fruits with three different pungency levels non-pungent, mild, and highly pungent, **b** on control diet and on chili fruits for 7 days and transferred to a regular artificial diet when exposed to the parasitoids, **c** a control diet and artificial diet mixed with three levels of synthetic capsaicin (no capsaicin, 20 and 200 ppm) and **d** a control diet and a capsaicin-spiked diet for 7 days before the parasitism and regular artificial diet when adding the parasitoids. For the control treatment, *S. latifascia* fed on maize leaf and regular artificial diet. Different letters indicate a significant difference between treatments (Chi-test, Tukey post hoc test with Bonferroni correction: *P* < 0.01). Sample sizes: Fig. 3a control = 10, non-pungent = 13, mild = 13 and highly pungent = 13; Fig. 3b, c and d, *N* = 5 for all treatments except for the control (*N* = 10)

The pungency level in fruits had a negative effect on parasitism rate (Fig. 3a, b). Parasitism rate was 30% lower on caterpillars reared on the highly pungent Habanero variety in the presence of fruits (Fig. 3a, *χ*^2^ = 57.196, d.f = 3, *P* = 0.06) and 80% lower on caterpillars reared on this same variety but exposed to the wasps without the fruits (Fig. 3b, *χ*^2^ = 11.506, d.f = 3, *P* = 0.003) than on caterpillars reared on the other two varieties. There was no significant difference on parasitism rate between mild and non-pungent treatments in the presence and absence of chili fruit (Fig. 3a, b). In the absence of the chili fruit when exposed to the wasps (Fig. 3b), parasitism rates for both mild and non-pungent treatments were as high as for the control, whereas when chili fruits were present, the parasitism rate for the same treatments was around 30% lower than the control (Fig. 3a).

Pungency level had no significant effect on the clutch size laid by *E. platyhypenae* on *S. latifascia* (Supplementary Fig. 2a, F_[3, 27]_ = 0.4695, d.f = 3, *P* = 0.70, Supplementary Fig. 2b, F_[3, 16]_ = 0.5711, d.f = 3, *P* = 0.64).

We found similar results for parasitoids when exposed to caterpillars reared on capsaicin-spiked diets. Parasitism rate was approximately 60% lower on larvae from the high-capsaicin diet (200 ppm) than on larvae from the other two treatments (no capsaicin) and 20 ppm (Fig. 3c, *χ*^2^ = 16.510, d.f = 3, *P* = 0.011). However, in the second experiment when larvae from the three spiked-diet treatments were switched to a non-spiked artificial diet and then exposed to parasitism, this protection was lost (Fig. 3d. *χ*^2^ = 6.5017, d.f = 3, *P* = 0.59).

No significant difference among treatments was found for clutch size (Supplementary Fig. 2c, F_[3, 15]_ = 3.037, d.f = 3, P = 0.061; Supplementary Fig. 2d, F_[3, 20]_ = 1.1686, d.f = 3, *P* = 0.34).

### Quantification of capsaicinoids in the haemolymph of *S. latifascia*

**Fig. 4:**
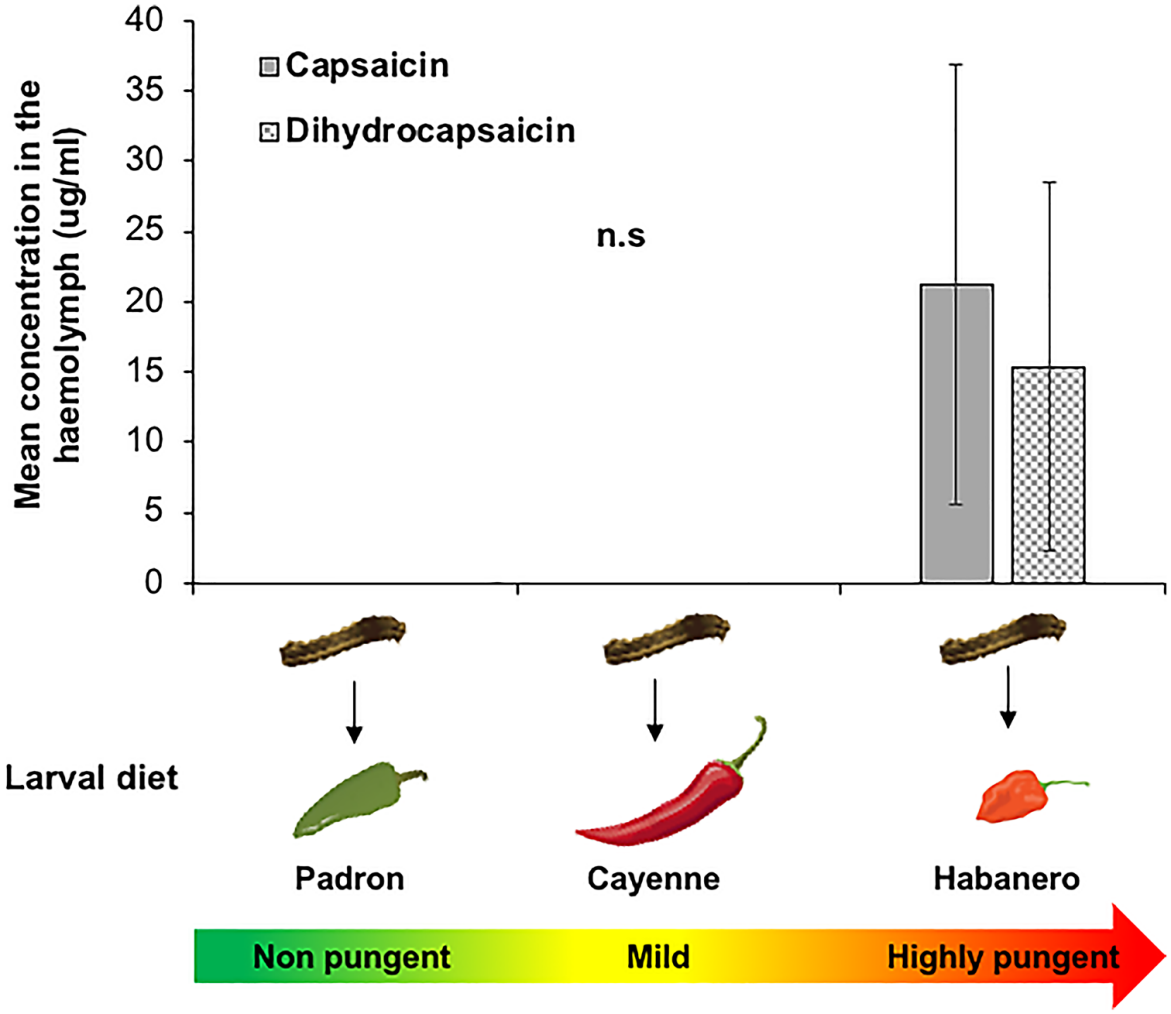
Mean (± SEM) capsaicinoids content (mg/g of DW) in the haemolymph of *Spodoptera latifascia* larvae when feeding on three chili varieties, Padron, Cayenne and Habanero. (F-test: *P* > 0.01, *N* = 5)

Capsaicinoids analysis in the haemolymph revealed that when caterpillars of *S. latifascia* were fed on habanero fruits, the levels of capsaicin and dihydrocapsaicin were 21 and 15 times, respectively, higher than when fed on fruits of the two other varieties (Fig. 4, capsaicin F_[2,12]_ = 1.826, *P* = 0.20 and dihydrocapsaicin; F_[2,12]_ = 1.3937, *P* = 0.28). Despite these very clear differences, the results were not significant most likely due to the high variability among larvae in the amount of placenta consumed, as capsaicinoids are mostly concentrated in this tissue and not in the rest of the fruit.

## Discussion

The aim of our study was to investigate the effect of capsaicin in domesticated chili peppers on a tritrophic interaction with the generalist herbivore *S. latifascia* and its ectoparasitoid *E. platyhypenae.* Overall, our results reveal that capsaicin had a negative effect on both insects, particularly at high concentrations. Indeed, we found that when larvae of *S. latifascia* were reared on pungent chili varieties, they had lower larval weight, reduced pupation, and lower adult emergence rates than when caterpillars were reared on non-pungent varieties. Similar results were found when caterpillars where reared on capsaicin-spiked and control diets. The negative effects of capsaicin subsequently affected the third trophic level by reducing the parasitism of caterpillars when these were reared on fruits or diet.

To date, all published studies on the effects of capsaicin on insects have focussed only on herbivores and most of these have used artificial diet or ground chili powder and not fresh fruits. These studies have mainly examined the benefits of capsaicin as a pesticide. For example, Cowles et al. (1989) found that oviposition of the onion fly (*Delia antiqua*) (Diptera: Anthomyiidae) was reduced by 99.8% and 95%, respectively, by applying both chili powder and synthetic capsaicin to artificial diet. Interestingly, despite capsaicinoids being naturally absent in leaves and vegetative organs of chili plants (Estrada et al. 2002), they are still effectively used as biopesticides against sucking insects attacking chili leaves, such as the green aphid *Myzus persicae* Sulz (Hemiptera: Aphididae) (Edelson et al. 2002; Koleva-Gudeva et al. 2013) or whiteflies, a major pest of pepper crops (Greer 2000). However, their mode of action remains unclear.

By using fresh chili fruits, we were able to assess the effect of natural capsaicin on the second and third trophic levels. Alternatively, the capsaicin-spiked diet allowed us to isolate the effect of capsaicin from other potential effects of the chili fruits on the insects. However, some differences were observed when using these two types of diets, particularly for the herbivore, where the negative effects of capsaicin were more evident when fed on fruits. We found that pungency level in fruits had a negative effect of on the parasitism both when the fruit was present or absent. However, with diet this effect was only evident when the spiked-capsaicin diet was removed (Fig. 3d) as compared to when it was still present during parasitism (Fig. 3c). It is possible that due to a lower amount of capsaicin in diet as compared to fruits, the lasting effects inside the host were shorter and parasitoids were not able to perceive it. Due to the high capsaicin content in the fruits, we could not mimic the exact levels because of the high toxicity we experienced while manipulating the pure synthetic capsaicin. Therefore, we would expect that the effect would be stronger if we increase the capsaicin content levels equivalent to those found in the fruits.

It is expected that the effects of capsaicin will be different on generalist than specialist herbivores. For instance, larval growth of the generalist herbivores *Spodoptera frugiperda*, *Heliothis virescens* and *Helicoverpa zea* (Lepidoptera: Noctuidae) was slower when fed with capsaicin-spiked diet, while the growth and survival of larvae of the tobacco budworm (*Helicoverpa assulta*) (Lepidoptera: Noctuidae)*,* a specialist on Solanaceae, was not affected (Ahn et al. 2011a). The latter is considered to be one of the few insect herbivores capable of feeding on hot pepper fruits (Baek et al. 2009) and able to detoxify these secondary metabolites (Ahn et al. 2011b). Another species known to feed on chili pepper is the pepper weevil, *Anthonomus eugenii* (Coleoptera: Curculionidae), a specialist primarily on fruits of *Capsicum* spp., but able to feed on other nightshade plants (e.g. eggplants) (Rodriguez-Leyva 2006). This beetle is known to feed on highly pungent chili varieties such as Habanero and Scotch Bonnet (Seal and Martin 2016). Adults lay eggs on flower buds and complete their development inside the fruits (Riley and Sparks Jr 1995). Both larvae and adults were observed feeding on the fruit’s placenta (Chabaane, personal observation) where capsaicinoids are concentrated (Fujiwake et al. 1982). It is assumed that both larvae and adults of this species can handle the spiciness, but the mechanism remains unclear. In our study, even though *S. latifascia* is a generalist, caterpillars were able to tolerate diet and fruits with medium levels of capsaicin. We found that while feeding, caterpillars can sequester capsaicin in the haemolymph, but only when they feed on the highly pungent varieties (Fig. 4). However, when exposed to lower levels of capsaicin, sequestration did not occur, or the levels were under the detection limit. It is possible that caterpillars are able to detoxify or excrete this secondary metabolite when present at low levels as it has been shown for other herbivores exposed to nicotine, also an alkaloid (Barbosa et al. 1986).

The effect of capsaicin on *S. latifascia* was stronger than on its parasitoid *E. platyhypenae*. For the parasitoid, we found differences among treatments only for parasitism rate but not for clutch size. Yet, as the parasitoid larvae feed on the host’s haemolymph (Coudron et al. 1990; Nakamatsu and Tanaka 2003) where the capsaicin can be found, we could expect to find stronger effects of capsaicin on the parasitoid larval and adult stages. Thus, further studies should focus beyond the oviposition response of the parasitoid and examine the effects of capsaicin throughout parasitoid development, adult survival, size, and sex ratio. Moreover, parasitism rate was also reduced when Habanero fruits were replaced by no-capsaicin diet during the exposure to wasps. This was probably caused by the capsaicinoids accumulated in the haemolymph. However, it remains to be investigated how long this accumulation will last, and the subsequent effects on the parasitoid once the exposure to capsaicin is stopped. It would also be interesting to test the effects of capsaicin on other natural enemies with different life history strategies and feeding modes such as endoparasitoids and predators.

Recently, a growing number of studies have examined the relationship between plant chemical defence as a result of domestication and insect performance (Chen et al. 2015; Whitehead et al. 2017). It is often found that lower chemical defence results in increased performance, but there are also many exceptions to this pattern (Shlichta et al. 2018; Turcotte et al. 2014). Chili pepper offers a unique model to examine this relationship, since we have varieties selected for lower capsaicinoid content than the wild chiltepin, but also varieties that were selected for much higher pungency levels (Scoville 1912). In another study, we found that the capsaicinoid levels in chiltepin fruits collected from different populations along the Pacific coast of the state of Oaxaca, Mexico, mainly ranged between the contents detected on Habanero and Cayenne varieties (Chabaane et al., unpublished results). Thus, we would expect that the performance of *S. latifascia* larvae on wild chili fruits would be intermediate compared to the highly pungent and mild varieties.

Here, we focussed on the effects of capsaicin, the main plant trait targeted during varietal selection of chili pepper (Paran and van der Knaap 2007). Yet, the domestication syndrome of *Capsicum* species includes other traits such as germination rate, fruit colour, position and size, foliar and phenological traits (Pickersgill 2016). It is likely that some or most of these traits will also affect insect choice and performance. Moreover, some of these traits might be correlated to the level of pungency in fruits. For example, Taiti et al. (2019) found that volatile organic compound (VOC) emissions from fresh chili fruits were correlated with their spiciness. It is known that parasitoids use plant volatiles to locate their hosts (Turlings et al. 1990; Vet and Dicke 1992). Therefore, future studies should investigate the influence of multiple domesticated traits on tritrophic interactions of this important crop.

Our results also offer further insight into alternative strategies for pest management in chili pepper, one of the top ten vegetable crops in the world (FAOSTAT 2020). The use of mixed varieties in agriculture has been shown to reduce pest pressure by slowing the spread of insects via resistant varieties acting as barriers (Barot 2017). For example, Abdala‐Roberts et al. (2015) showed that mixed genotypes of *Capsicum chinense* reduced damage of the leaf mining fly (*Liriomyza trifolii*) (Diptera: Agromyzidae) by 25% as compared to monoculture. They suggest that plant genotypic diversity of their varieties (e.g. plant size, architecture, flowering phenology, and fruit size) played an important role in reducing insect attack. The importance of genotype diversity in chili peppers was also reported to decrease infestation by whitefly (*Bemisia tabaci*) (Hemiptera: Aleyrodidae) and yellow mite (*Polyphagotarsonemus latus*) (Acari: Tarsonemidae) (Datta and Chakraborty 2013), as well as black aphid (*Aphis craccivora*) (Homoptera: Aphididae) (Ofori et al. 2015) infestations on peppers. In this context, our results suggest that growing mixed chili varieties with different pungency levels, in addition to the use of parasitoids, might reduce pest pressure by generalist herbivores. Therefore, chili pepper farmers should include these practices in their IPM (Integrated pest management) programmes to optimize pest control.

In conclusion, our study represents pioneering work regarding the effect of natural capsaicin on herbivores and the third trophic level. In the future, this knowledge could also be important for other crops, such as hot tomato, which could be developed by activating the inactive capsaicinoid biosynthesis pathway naturally present in this Solanaceous crop (Naves et al. 2019). Moreover, as chili peppers originate and were domesticated in Mesoamerica, by studying its interactions with native insects from this region, our results could provide insight into the selective pressures that have contributed to the crop’s phenotypic diversity and the relationship with its wild ancestor.

## Author contributions

YC and BB conceived and designed the research. YC performed experiments. YC and CMA developed the methods. YC and CMA analysed the data. GG performed the chemical analyses. YC and BB wrote the first drafts. All authors contributed to the last versions of the manuscript.

## Supplementary Information

Below is the link to the electronic supplementary material.

Supplementary file1 (PDF 3912 KB)
