# Supplementary material for: Altered capsaicin levels in domesticated chili pepper varieties affect the interaction between a generalist herbivore and its ectoparasitoid
Source: J Pest Sci (2004). 2021 Jun 25;95(2):735–47. doi: 10.1007/s10340-021-01399-8 (PMC8860780; doi:10.1007/s10340-021-01399-8)
Supplement: Supplementary file 1 — Supplementary file1 (PDF 3912 KB) [file 10340_2021_1399_MOESM1_ESM.pdf]

ALTERED CAPSAICIN LEVELS IN DOMESTICATED CHILI PEPPER VARIETIES AFFECT THE INTERACTION BETWEEN A GENERALIST HERBIVORE AND ITS ECTOPARASITOID

YOSRA CHABAANE[1], CARLA MARQUES ARCE[2], GAËTAN GLAUSER[3] AND BETTY BENREY[1*]

[1]Laboratory of Evolutionary Entomology, Institute of Biology, University of Neuchâtel, Rue Emile-Argand 11, 2000 Neuchâtel, Switzerland

[2]Fundamental and applied research in chemical ecology, Institute of Biology, University of Neuchâtel, Rue Emile-Argand 11, 2000 Neuchâtel, Switzerland

[3]Neuchâtel platform of analytical chemistry, Institute of Chemistry, University of Neuchâtel, Rue Emile-Argand 11, 2000 Neuchâtel, Switzerland

*Author for correspondence (e-mail: betty.benrey@unine.ch)

Journal of Pest Science

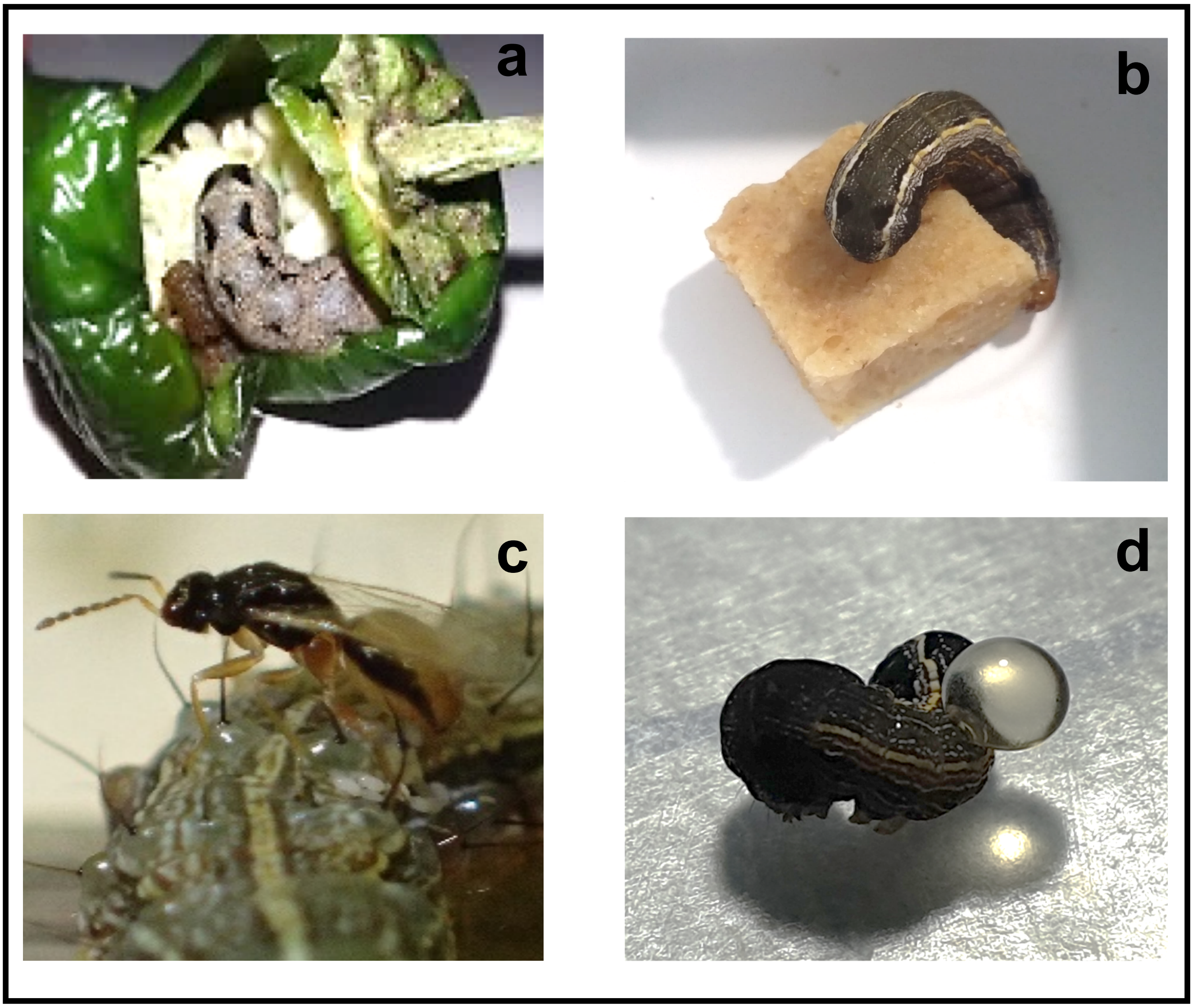

Supplementary Fig. 1 *Spodoptera latifascia* caterpillars feeding on chili fruits (**a**), on artificial diet (**b**), its female parasitoid *Euplecterus platyhypenae* (**c**) and its haemolymph extracted from the dorsal part of the thorax (**d**)

Supplementary Table 1 Mean pupal weight (mg) of *Spodoptera latifascia* feeding on chili varieties with three different pungency levels: non pungent (Padron), mild (Cayenne) and highly pungent (Habanero) (F-test: $P > 0.05$, Sample sizes = Number of pupa)

Pungency level	Variety name	Mean pupal weight (mg)	Standard Error	Number of pupa
Non pungent	Padron	542.81	14.37	15
Mild	Cayenne	568,43	44.49	4
Highly pungent	Habanero	539,16	74.16	5

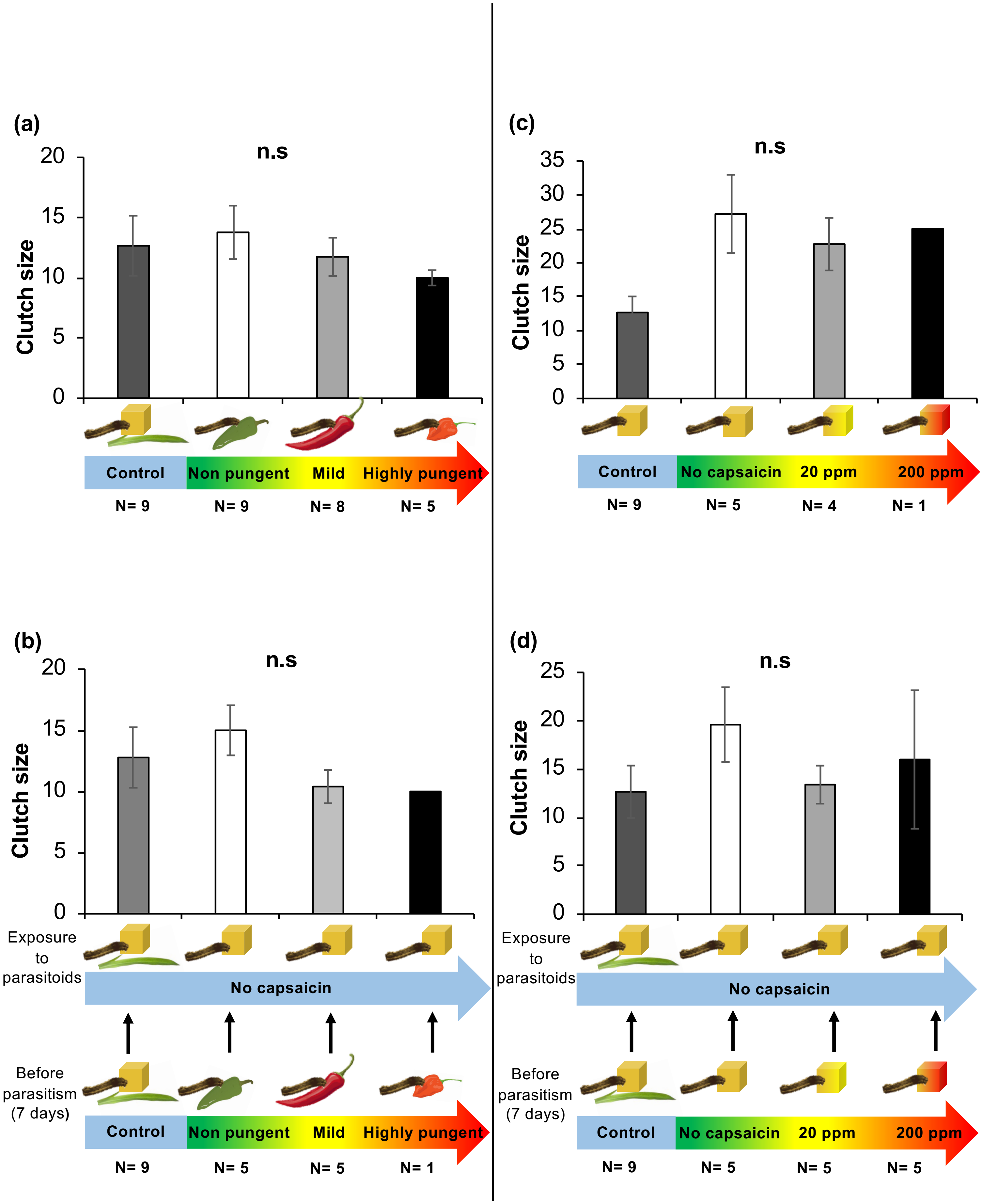

Supplementary Fig. 2 Clutch size (Mean (±SEM) number of parasitoid eggs laid on a caterpillar) of *Euplectrus platyhypenae* on *Spodoptora latifascia* caterpillars feeding on **(a)** control diet and on chili fruits with three different pungency levels non pungent, mild, and highly pungent, **(b)** on control diet and on chili fruits for 7 days and transferred to a regular artificial diet when exposed to the parasitoids, **(c)** on control diet and artificial diet mixed with three levels of synthetic capsaicin (no capsaicin, 20 and 200 ppm) and **(d)** on control diet and a capsaicin-spiked diet for 7 days before the parasitism and regular artificial diet when adding the parasitoids. For the control treatment, *S.latifascia* fed on maize leaf and regular artificial diet. No significant difference between treatments (F-test: $P > 0.05$). Sample sizes are indicated directly in the figures (N = number of parasitized larva)
